# Stem Cell-Engineered Nanovesicles Exert Proangiogenic and Neuroprotective Effects

**DOI:** 10.3390/ma14051078

**Published:** 2021-02-25

**Authors:** Han Young Kim, Suk Ho Bhang

**Affiliations:** 1 Biomedical Research Institute, Korea Institute of Science and Technology, Seoul 02792, Korea; hy0408@kist.re.kr; 2 School of Chemical Engineering, Sungkyunkwan University, Suwon 16419, Korea

**Keywords:** angiogenesis, mesenchymal stem cells, nanovesicles, neuroprotective effect

## Abstract

As a tissue regeneration strategy, the utilization of mesenchymal stem cells (MSCs) has drawn considerable attention. Comprehensive research using MSCs has led to significant preclinical or clinical outcomes; however, improving the survival rate, engraftment efficacy, and immunogenicity of implanted MSCs remains challenging. Although MSC-derived exosomes were recently introduced and reported to have great potential to replace conventional MSC-based therapeutics, the poor production yield and heterogeneity of exosomes are critical hurdles for their further applications. Herein, we report the fabrication of exosome-mimetic MSC-engineered nanovesicles (MSC-NVs) by subjecting cells to serial extrusion through filters. The fabricated MSC-NVs exhibit a hydrodynamic size of ~120 nm, which is considerably smaller than the size of MSCs (~30 μm). MSC-NVs contain both MSC markers and exosome markers. Importantly, various therapeutic growth factors originating from parent MSCs are encapsulated in the MSC-NVs. The MSC-NVs exerted various therapeutic effects comparable to those of MSCs. They also significantly induced the angiogenesis of endothelial cells and showed neuroprotective effects in damaged neuronal cells. The results collectively demonstrate that the fabricated MSC-NVs can serve as a nanosized therapeutic agent for tissue regeneration.

## 1. Introduction

Owing to their unique properties, mesenchymal stem cells (MSCs) have attracted attention for applications in the treatment of various diseases. In particular, MSCs have wide application prospects in the restoration of injured tissue [1,2,3]. Numerous preclinical and clinical trials utilizing MSCs have been accomplished with significant clinical outcomes [4,5]. It is reported that there are over 1138 registered clinical trials worldwide using MSCs as a therapeutic agent, which is a significant increase from the 220 registered in 2012 [6]. However, accumulating evidence strongly suggests that MSCs do not replace damaged cells in the injected region. The poor survival and fast clearance of transplanted MSCs indicate that the major therapeutic effects of MSCs in injured tissues are attributed to paracrine factors secreted by MSCs [7].

A recent study reported that the key factor playing a major role in the therapeutic mechanism of MSCs is paracrine signaling from MSCs, including growth factors and extracellular vesicles such as exosomes [8]. Exosomes are cell-derived extracellular vesicles, with sizes ranging from 50 to 150 nm. These contain genes and proteins originating from donor cells [9]. Importantly, exosomes derived from MSCs have recently emerged as novel therapeutic agents for the treatment of various diseases, including myocardial infarction [10] and those related to the central nervous system (CNS) [11,12,13] and muscles [14]. Thus, exosomes have been proposed as a strategy for “cell-free regenerative medicine.” Their unique properties of stability, biocompatibility, and low immunogenicity have prompted research into their potential as therapeutic delivery agents [15], not only for animal studies but also for human clinical trials [16]. However, although MSC exosomes have great potency for the treatment of various diseases, their poor production yield is a critical hurdle for their further application. Most mammalian cells release low quantities of exosomes, and the purification of exosomes is challenging, which results in a low yield [17,18]. Especially for MSCs, very small amounts of exosomes are produced [19]. This indicates that large-scale and repetitive cell culture is required to obtain a sufficient supply of exosomes for further studies. It is also reported that cells release distinct exosome subpopulations that elicit differential effects on recipient cells [20].

Thus, in this study, we utilized cell-derived nanovesicles (NVs), which are exosome mimetic. Exosome-mimetic NVs can be produced by subjecting cells to serial extrusion and can be purified using density-gradient ultracentrifugation [21]. The desired size of NVs can be obtained by modulating the size of the filter pore used during serial extrusion. Importantly, because the isolation of NVs does not require ultracentrifugation of the conditioned medium, which is used for the isolation of natural exosomes, the production yield of exosome-mimetic NVs is significantly higher than that of natural exosomes (~100-fold higher). Furthermore, it is known that NVs contain larger amounts of intracellular contents than exosomes [22]. In this context, we fabricated MSC-derived NVs (MSC-NVs). We evaluated the morphology and size of MSC-NVs and compared their gene and protein expressions with those of the parent MSCs. The therapeutic mechanisms of MSCs in the treatment of heart or CNS injuries rely on their proangiogenic effect on endothelial cells [23,24] and neuroprotective effect on injured neurons [25,26]. In addition to MSCs, MSC-derived exosomes are reported to exert those effects [27]. Therapeutic applications of cell-derived NVs were recently reported. NVs derived from hepatocyte regenerated injured liver in vivo [28] and long noncoding RNA H19-containing NVs accelerated the healing process of diabetic wounds [29]. Previous studies showed that MSC-NVs fabricated by extrusion can ameliorate sepsis [30] or accelerate skin wound closure [31], suggesting MSC-NVs can be used as an alternative therapeutic agent that can replace MSCs or MSC exosomes. However, there is a lack of studies with comprehensive in vitro analysis demonstrating the proangiogenic and neuroprotective effects of MSC-NVs. In this context, herein, we investigate whether the fabricated MSC-NVs show proangiogenic and neuroprotective effects.

## 2. Materials and Methods

### 2.1. Cell Culture

The human bone marrow derived MSCs used in this study were purchased from Lonza (Basel, Switzerland). MSCs were cultured in Dulbecco’s modified Eagle’s medium (Gibco BRL, Gaithersburg, MD, USA) supplemented with 10% (*v/v*) fetal bovine serum (FBS), penicillin (100 units/mL), and streptomycin (100 μg/mL). Cells were cultured and maintained at the passage numbers from 5 to 7. Human umbilical vein endothelial cells (HUVECs, Lonza) were cultured in 200PRF (Gibco BRL) supplemented with a low-serum growth supplement (Gibco BRL). PC12 cells purchased from Paragon Biotech (Baltimore MD, USA) were cultured in RPMI 1640 (Gibco BRL) supplemented with 7.5% (*v/v*) FBS, 7.5% (*v/v*) horse serum, and 1% (*v/v*) penicillin/streptomycin. The culture medium was exchanged every three days. Nerve growth factor (100 ng/ mL, Sigma, St. Louis, MO, USA) was used for neuronal differentiation of PC12 cells.

### 2.2. Fabrication and Purification of MSC-NVs

As soon as the cultured MSCs reached ~90% confluence in 150 mm^2^ culture dishes, the cells were detached from the dishes by a cell scraper and centrifuged at 300× *g* for 5 min. Cell pellets were collected, and the cells were resuspended in PBS at a concentration of 2 × 10^6^ cells/mL. Then, suspended MSCs were extruded through 10 μm, 5 μm, 1 μm, and 400 nm pore-sized membrane filters using a mini extruder (Avanti Polar Lipids, Birmingham, AL, USA). Cells were extruded five times in each membrane filter. Extruded MSC-NVs were then purified by density-gradient ultracentrifugation. A step gradient was formed using OptiPrep (Axis-shield, Dundee, UK). OptiPrep (10% (*v/v*)) was overlaid on top of 50% (*v/v*) OptiPrep, and extruded MSC-NVs were added on top of the tube. After ultracentrifugation at 100,000× *g* for 2 h, the fraction between 50% and 10% OptiPrep was collected and ultracentrifuged again at 100,000× *g* for 2 h. The pellet was resuspended in PBS and filtered using a 0.22 μm syringe-filter (Merck Millipore, Billerica, MA, USA). Fabricated MSC-NVs were aliquoted and stored at −80 °C until needed.

### 2.3. Characterization of MSC-NVs

The fabricated MSC-NVs were observed using transmission electron microscopy (TEM, JEOL, JEM-2100F, Tokyo, Japan). For TEM observation, MSC-NVs were loaded on thin formvar/carbon film-coated 200 mesh copper grids and were negatively stained using 1% uranyl acetate solution. The MSC-NVs were visualized using LIBRA 120 (Carl Zeiss, Jena, Germany). The hydrodynamic size of the fabricated MSC-NVs was measured using a nanoparticle tracking analysis (NTA) system. By using Nanosight (LM10, Salisbury, UK), the particle numbers and the size of the MSC-NVs were determined. 

### 2.4. Gene and Protein Expression Analysis of MSC-NVs

Genes or proteins inside the MSC-NVs were extracted with either TRIzol (Life Technologies, Carlsbad, CA, USA) or cell lysis buffer (Cell Signaling Technology, Danvers, MA, USA), according to the manufacturer’s instructions. Total RNAs inside the MSC-NVs were collected using TRIzol, and the concentration of isolated RNA was measured using the NanoDrop spectrophotometer (Thermo Fisher Scientific, Waltham, MA, USA). The concentration of proteins extracted from the MSC-NVs was measured by the Bradford assay using the Bradford reagent (Sigma). For the proteomic profiling of MSCs and MSC-NVs, Coomassie blue staining was performed. Briefly, proteins isolated from MSCs or MSC-NVs were mixed with LDS sample buffer (Life Technologies) and loaded on 10% (w/v) SDS/polyacrylamide gel. After 100 min of electrophoresis at 80V, gels were stained using PageBlue staining solution (Thermo Fisher Scientific). Stained gels were then visualized using an iBright FL1000 imaging system (Invitrogen, Carlsbad, CA, USA). For Western blot analysis, proteins in the gel were transferred to an Immobilon-P membrane (Merck Millipore, Burlington, MA, USA) using the Trans-Blot^®^ SD Semi-Dry Electrophoretic Transfer Cell (Bio-Rad, Hercules, CA, USA) for 50 min at 20V. After blocking with 5% skim milk, the membranes were probed with primary antibodies overnight. All antibodies were purchased from Abcam (Cambridge, CA, USA). The membranes were intensively washed with TBS-T and incubated with a HRP-linked goat antirabbit secondary antibody or HRP-linked horse antimouse secondary antibody for 1 h at room temperature. Captured proteins were developed using a chemiluminescence detection system. Reverse transcriptase–polymerase chain reaction (RT-PCR) was performed using cDNA synthesized from the total RNAs. Amplified cDNA was mixed with a loading buffer and loaded on 1.5% agarose gel, followed by gel electrophoresis at 75V for 30 min. Primers used for RT-PCR are listed as follows: CD29 forward: CCA CAG ATG CCG GGT TTC ACT, CD29 reverse: CCT TCG CTT TGG TCA ATT. CD34 forward: TGA AAA AGC TGG GGA TCC TAG, CD34 reverse: TCC CAG GTC CTG AGC TAT AGC. CD44 forward: GAC ACA TAT TGC TTC AAT GCT T, CD44 reverse: GAT GCC AAG ATG ATC AGC CAT. GAPDH forward: CCA CTC CTC CAC CTT TGA, GAPDH reverse: ACC CTG TTG CTG TAG CCA.

### 2.5. In Vitro Evaluation of the Proangiogenic Effects of MSC-NVs

To assess the angiogenesis of HUVECs after treatment of MSC-NVs, their cell migration, proliferation, and tube formation were evaluated. MSC-NVs (4 mg/mL) were diluted and treated at a concentration of 40 μg/mL. For observation of the cellular internalization of the NVs into HUVECs, 3,3′-dioctadecyloxacarbocyanine perchlorate (DiO) was used to label the NVs. After 2 h of treatment, the cells were observed with fluorescence microscopy (Bx51, Olympus, Tokyo, Japan). For the migration assay, HUVECs were seeded on 6-well plates, and a confluent monolayer was scratched with a pipette tip. After incubation with 1 mL of serum-free medium containing either PBS (1 μL) or MSC-NVs (1 μL, 40 μg/mL), optical microscopy images of HUVECs were obtained at 0 and 24 h. The treatment of 1% (*v/v*) PBS served as a negative control. The cell-free area was quantitatively measured from three images taken from each well at each time point, using ImageJ software (*n* = 3 wells per group; total of 9 images). The proliferation of HUVECs was also determined after treatment with increasing doses of MSC-NVs. HUVECs were treated with a cell medium containing 1% (*v/v*) PBS or 5, 10, 20, and 40 μg/mL of MSC-NVs. At 0, 24, 48, and 72 h after treatment, the cell growth of HUVECs was determined using the CCK-8 assay kit (EZ-Cytox, DoGenBio, Seoul, Korea). To investigate the tube formation efficacy of HUVECs upon treatment with MSC-NVs (40 μg/mL), the cells were seeded on Matrigel-coated 6-well plates. At 0 and 8 h after the treatment of cell medium containing either 1% (*v/v*) PBS or MSC-NVs (40 μg/mL), microscopy images of HUVECs were obtained. The number of tubes and junctions were counted and quantified from five images taken from each well using ImageJ software with the Angiogenesis Analyzer plugin tool (*n* = 3 wells per group, total 15 images).

### 2.6. In Vitro Assessments of the Neuroprotective Effects of MSC-NVs

To determine the neuroprotective effects of the MSC-NVs, neuronally differentiated PC12 cells were used. For the neuronal differentiation of PC12 cells, the cells were incubated with a cell medium containing NGF (100 ng/mL) and replenished every 2 days. After 7 days of differentiation, PC12 cells were treated with a cell medium containing either 1% (*v/v*) PBS or MSC-NVs (40 μg/mL) for 16 h. Next, the cells were washed three times with PBS and incubated with a cell medium containing a high concentration of LPS (1 μg/mL, Sigma). Then, the cells were incubated under hypoxic conditions (1% oxygen). After 24 h of incubation, the cells were washed three times with PBS and immediately double-stained with annexin V-FITC (BioLegend, San Diego, CA, USA) and propidium iodide (PI; BioLegend), followed by incubation at 4 °C for 30 min in the dark. Annexin-V-positive (excitation: 494 nm, emission: 519 nm) and PI-positive (excitation: 536 nm, emission: 617 nm) PC12 cells were determined with flow cytometry using FACSAria II (BD Bioscience, San Jose, CA, USA). The collected results were analyzed and quantified using FlowJo software (BD Bioscience), with no gating. Fluorescence compensation was applied to reduce the crosstalk between FITC and PI. At the same time point, cell viabilities of PC12 cells with different treatments were evaluated using the CCK-8 assay. Additionally, the mRNA expression of *Bcl-2* and *Bax* in PC12 cells was examined using quantitative real-time polymerase chain reaction (qRT-PCR). For qRT-PCR, FAST SYBR Green PCR master mix (Applied Biosystems) was used, and mRNA expression was determined using StepOnePlus RT-PCR system (Applied Biosystems, CA) with amplifications with 40 cycles at 95 °C for 10 s, at 60 °C for 15 s, and at 72 °C for 30 s. Primers used for qRT-PCR are listed as follows: *Bax* forward: TCG CAG AGG ATG ATT GCT GA, *Bax* reverse: GAT CAG CTC GGG CAC TTT AG. *Bcl-2* forward: GCT ACG AGT GGG ATA CTG, *Bcl-2* reverse: GTG TGC AGA TGC CGG TTC.

### 2.7. Statistical Analysis

The data acquired in this study are presented as the mean ± standard deviation (SD). Values are representative of three independent experiments with three or more samples per group. MSC-NVs isolated from three different donor MSCs were used in each independent experiment. The *p*-values were calculated by one-way analysis of variance (ANOVA) or *t*-test with Tukey’s post hoc test. Graphs and plots were generated using GraphPad Prism software. Values of *p* < 0.05 were considered statistically significant.

## 3. Results and Discussion

### 3.1. Fabrication and Characterization of MSC-NVs

**Figure 1:**
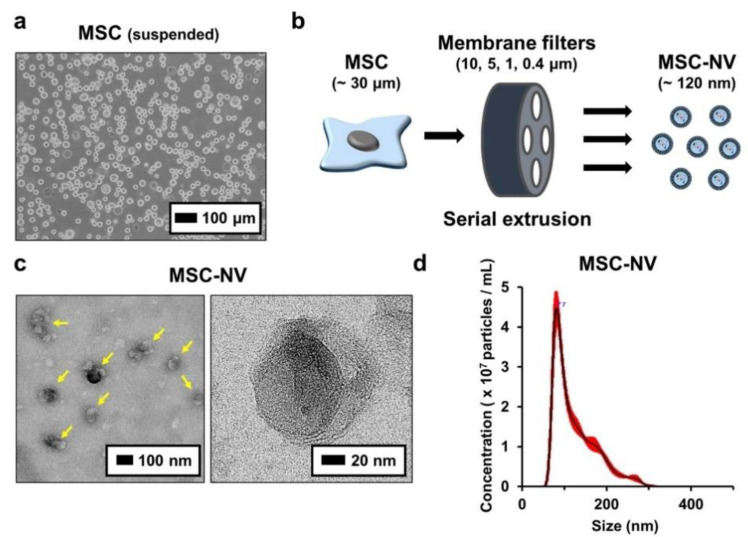
(**a**) Morphological observation of human bone marrow derived mesenchymal stem cells (MSCs). Suspended MSCs were observed using optical microscopy. (**b**) Schematic illustration showing the fabrication of MSC-engineered nanovesicles (MSC-NVs) by serial extrusion of MSCs. (**c**) TEM image of MSC-NVs. The yellow arrows in the left panel indicate MSC-NVs. (**d**) Size distribution of isolated MSC-NVs, determined by nanoparticle tracking analysis (NTA).

To fabricate MSC-NVs, human bone marrow derived MSCs with passage numbers of 5–7 were used. Bone-marrow-derived MSCs were detached and suspended (Figure 1a). Then, MSC-NVs were fabricated by serial extrusion of MSCs, as shown in Figure 1b. It is known that during cell extrusion, the cell membrane undergoes deformation, and cell components such as mRNA, DNA, and proteins start to leak out. However, the lipid bilayer fragments immediately self-assemble and encapsulate these fragments [22]. MSCs were sequentially extruded through 10, 5, 1, and 0.4 μm pore-sized membrane filters. After purification by density-gradient ultracentrifugation, the collected MSC-NVs were observed using TEM (Figure 1c). MSC-NVs (yellow arrows in the left panel) exhibited a well-defined spherical shape, which is similar to the morphology of MSC-derived exosomes [32]. Next, the size of the MSC-NVs was measured using a nanoparticle tracking analysis (NTA) system (Figure 1d). The obtained MSC-NVs exhibited a mean size of 121.8 ± 1.6 nm, which is also similar to the size of MSC-derived exosomes. These results demonstrate that the MSC-NVs were successfully isolated and that their morphology and size were very similar to those of MSC-derived exosomes.

### 3.2. MSC-NVs Contain Proangiogenic and Neuroprotective Growth Factors

**Figure 2:**
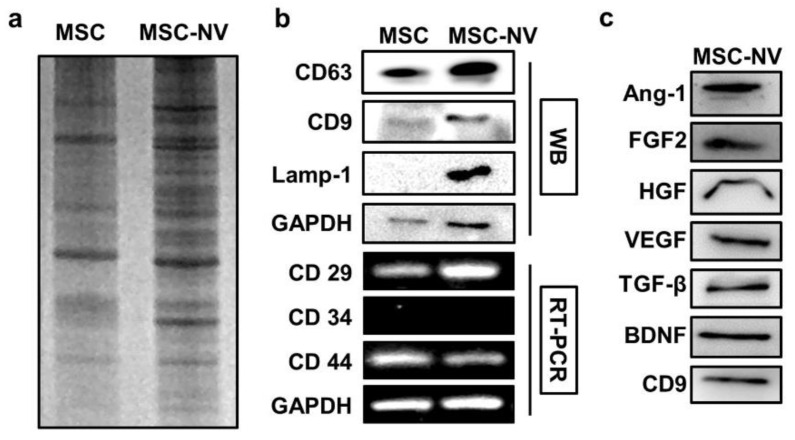
(**a**) Coomassie blue gel of lysates from MSCs and MSC-NVs, showing different patterns after the fabrication of MSC-NVs. (**b**) Western blot (WB) analysis of exosome markers (CD63, CD9, and Lamp-1), and reverse transcriptase–polymerase chain reaction (RT-PCR) analysis of MSC markers (CD29+, CD34−, and CD44+) in MSCs and MSC-NVs. (**c**) Evaluation of various growth factors and neurotrophic factors contained inside MSC-NVs.

To gain insights into the MSC-NVs and investigate how their components are similar or different compared to those of the parent MSC, the gene and protein expressions of MSC-NVs were evaluated. We first evaluated the protein expression patterns of MSCs and MSC-NVs. Equivalent amounts of proteins collected from MSCs or MSC-NVs were loaded on SDS gel and separated by electrophoresis, followed by Coomassie blue staining (Figure 2a). Consistent with previous reports, the visualization of MSC and MSC-NVs proteins revealed distinct protein expressions in MSC-NVs compared to their parent MSCs [33]. Based on the aforementioned experimental results that MSC-NVs exhibit a similar shape and hydrodynamic size compared to those of natural exosomes, we next determined and compared the exosomal enriched proteins in MSC-NVs. As evaluated by Western blot analysis, MSCs strongly expressed typical exosomal proteins such as CD63, CD9, and Lamp-1, whereas MSCs exhibited weak band signals (Figure 2b). This indicates that the fabricated MSC-NVs are exosome like. To investigate whether the MSC-NVs contained MSC-specific markers (CD29+, CD34−, and CD44+), we performed reverse transcriptase–polymerase chain reaction (RT-PCR). Agarose gel electrophoresis showed that the MSC-NVs contained MSC-specific markers originating from the parent MSCs. Because various therapeutic growth factors expressed in MSCs mainly contribute to the therapeutic effect of MSCs [34], we next determined whether those therapeutic growth factors exist inside the fabricated MSC-NVs. The expressions of proangiogenic and neuroprotective proteins such as angiopoietin-1 (Ang-1), basic fibroblast growth factor (FGF2), hepatocyte growth factor (HGF), vascular endothelial growth factor (VEGF), transforming growth factor beta (TGF-β), and brain-derived neurotrophic factor (BDNF) in the MSC-NVs were assessed by Western blot analysis (Figure 2c). The results demonstrate that the MSC-NVs contain those therapeutic growth factors and neurotrophic factors.

### 3.3. Proangiogenic Effects of MSC-NVs on Endothelial Cells

**Figure 3:**
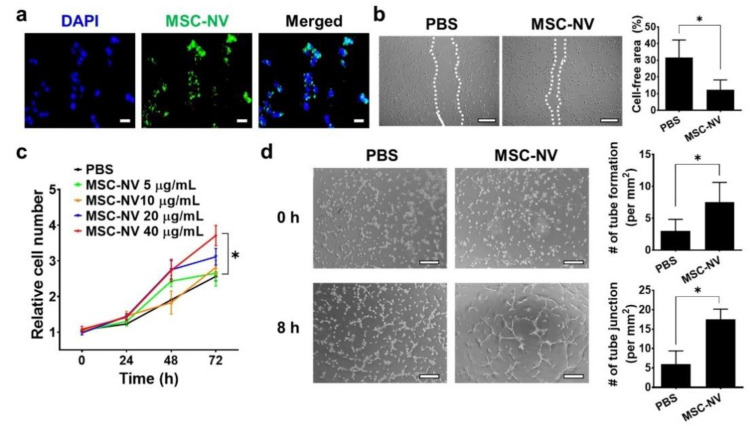
(**a**) Fluorescence microscopy image of human umbilical vein endothelial cells (HUVECs) treated with DiO (green)-stained MSC-NVs (40 μg/mL). Cells were observed at 2 h post-treatment of MSC-NVs. DAPI = nuclei, scale bar = 50 μm. (**b**) Migration of HUVECs treated with PBS or MSC-NVs (40 μg/mL) in serum-free medium at 24 h post-treatment. Scale bar = 100 μm. For quantitative analysis, the cell-free area was measured. *n* = 3, * *p* < 0.05. (**c**) Proliferation of HUVEC until 72 h after treatments with various concentrations of MSC-NVs (5–40 μg/mL). *n* = 5, * *p* < 0.05. (**d**) Tube formation of HUVECs at 0 h and 8 h after treatment with PBS or MSC-NVs (40 μg/mL). Tube formation and tube junctions were counted for quantification. Scale bar = 100 μm, *n* = 3, * *p* < 0.05. Three independent experiments were carried out, and the *p*-values were calculated by a two-tailed *t*-test.

Angiogenesis plays a pivotal role in the physiological tissue repair process [35]. It is essential for the formation of a new vascular network to supply sufficient oxygen and nutrients to the injured tissue. Based on our proteomic analysis, MSC-NVs contain various therapeutic growth factors such as Ang-1, FGF2, HGF, VEGF, and TGF-β, all of which are reported to effectively promote the angiogenesis of endothelial cells [36,37]. Thus, we next evaluated the proangiogenic effect of MSC-NVs using HUVECs. To determine the cellular internalization of MSC-NVs into HUVECs, 3,3′-dioctadecyloxacarbocyanine perchlorate (DiO), which emits green fluorescence, was used to stain the MSC-NVs (Figure 3a). Using fluorescence microscopy, we observed internalized MSC-NVs in HUVECs. Next, we investigated whether internalized MSC-NVs can enhance the migration of HUVECs. The scratch assay, which is typically used to assess cellular migration, was performed (Figure 3b). Immediately after scraping the monolayer of HUVECs in a straight line, the cells were incubated with MSC-NVs. At 24 h post-treatment, HUVECs treated with MSC-NVs exhibited significantly enhanced migration compared to that of PBS-treated cells, which served as a negative control. Quantitative measurement of the cell-free area demonstrated the significantly enhanced migration of HUVECs. We also investigated the effect of MSC-NVs on the proliferative behavior of HUVECs (Figure 3c). An increasing dose of MSC-NVs (5-40 μg/mL) was treated to HUVECs, and cell proliferation was evaluated by CCK-8 assay. After 72 h of treatment, the proliferation of HUVECs was significantly enhanced in a dose-dependent manner. Interestingly, MSC-NVs (40 μg/mL) showed a ~40% proliferation rate until 24 h. It is a significantly lower amount with a higher proliferative effect compared to a previous study using MSC-derived exosomes (100 μg/mL) that promoted a ~30% proliferation rate of HUVECs until 24 h [38]. In this study, we did not compare our finding with the MSC exosome, and further elucidation is required. A larger amount of proteins, including growth factors contained in artificial NVs, may contribute to the augmented proliferative effects of MSC-NVs [22]. To verify the proangiogenic potency of MSC-NVs, a tube formation assay was carried out (Figure 3d). Upon incubation with MSC-NVs, the capillary tube formation of HUVECs was determined at 8 h after incubation. In comparison with that observed in the PBS-treated group, HUVECs treated with MSC-NVs clearly showed capillary tube formation. Quantification analysis revealed that the number of tubes and tube junctions in MSC-NV-treated HUVECs was statistically higher than that observed in the negative control.

### 3.4. Internalized MSC-NVs Exert Neuroprotective Effects

**Figure 4:**
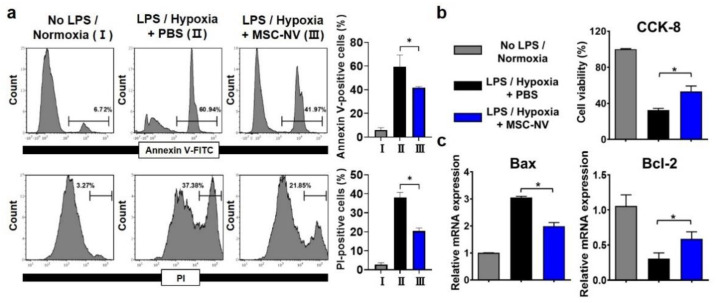
Apoptotic behavior of PC12 cells pretreated with MSC-NVs for 16 h and exposed to proinflammatory (LPS) and hypoxic (1% oxygen) conditions for 24 h. (**a**) Cells were double-stained with annexin V-FITC and propidium iodide (PI) and determined using flow cytometry. Quantitative analysis of flow cytometry was also carried out. *n* = 3, * *p* < 0.05. (**b**) Cell viability of PC12 cells measured by CCK-8 assay. *n* = 3, * *p* < 0.05. (**c**) mRNA expressions of Bax (proapoptotic) and Bcl-2 (anti-apoptotic) in PC12 cells, as evaluated by RT-PCR. *n* = 3, * *p* < 0.05. Three independent experiments were carried out, and the *p*-values were calculated by the one-way analysis of variance (ANOVA) with Tukey’s post hoc test.

In addition to promoting blood vessel formation, protecting neurons from cell death is a key factor for successive regenerative therapy [39]. After CNS injuries, neuronal cell death occurs due to severe inflammation and the depletion of oxygen [40]. Several previous reports suggest that the extracellular vesicles derived from MSCs exert neuroprotective effects [41,42]. Thus, we examined whether MSC-NVs can protect neurons from cell death. Neuronally differentiated PC12 cells were tested for the evaluation of the neuroprotective effects of MSC-NVs. Prior to induction of cell death, the PC12 cells were treated with either MSC-NVs or PBS. To induce neuronal apoptosis, we treated cells with high concentrations of lipopolysaccharide (LPS) and incubated them under hypoxic (1% oxygen) conditions, both of which are reported to induce high levels of cell apoptosis [42,43]. After 24 h, we assessed cell apoptosis using flow cytometry (Figure 4a). Cells were stained with annexin V and propidium iodide (PI). After incubating PC12 cells with LPS and in a hypoxic environment, severe cell apoptosis was observed. Annexin V-positive and PI-positive populations increased from ~6% to ~60% and from ~3% to 37%. However, pre-treatment with MSC-NVs resulted in a significant reduction in cell apoptosis. Annexin V-positive and PI-positive populations decreased to ~41% and ~21%. Next, we assessed the cell viability in the corresponding conditions by CCK-8 assay (Figure 4b). Although the treatment with LPS and incubation under hypoxic conditions resulted in a reduction of cell viability, pretreatment with MSC-NVs significantly increased the cell viability of PC12 cells. Evaluation by qRT-PCR showed that the expression of B-cell lymphoma-2 (Bcl-2) which is an apoptosis suppressor gene, is upregulated in MSC-NV-treated PC12 cells. In contrast, proapoptotic Bcl-2-associated X-protein (Bax) was downregulated compared to that in the control group.

## 4. Conclusions

This study demonstrates the fabrication of nanometer-sized MSC-NVs, which exert a proangiogenic effect on endothelial cells and neuroprotective effects on neurons. By serial extrusion of MSCs and density-gradient purification, we obtained MSC-NVs with a well-defined spherical shape and size similar to that of exosomes. The MSC-NVs expressed various exosome markers together with MSC-specific markers, indicating that they are exosome mimetic and contain MSC-derived genes and proteins. Importantly, we revealed that the MSC-NVs contain various types of therapeutic growth factors and neurotrophic factors that originate from the parent MSCs. Evaluated in vitro, the MSC-NVs enhanced the migration, proliferation, and tube formation of HUVECs. These results demonstrate the proangiogenic effect of MSC-NVs. Furthermore, the MSC-NVs significantly attenuated the apoptosis of neuronal cells. Experimental conditions such as high concentrations of LPS and incubation under hypoxic conditions caused neuronal cell death; however, the MSC-NVs exerted a neuroprotective effect and increased cell viability. Together, our results collectively demonstrate that MSC-NVs can serve as a novel therapeutic agent for tissue regeneration.

## Data Availability

The data presented in this study are available on request from the corresponding author.
